# Correlation analysis of serum PACAP-38, fibulin-3 and TEM1 levels with cardiac function and prognosis in chronic heart failure patients

**DOI:** 10.5937/jomb0-61365

**Published:** 2026-04-15

**Authors:** Yan Chen, Fanlong Zeng, Tianyun Gao, Bayi Hu, Mo Zhang, Zongliang Yu

**Affiliations:** 1 Affiliated Kunshan Hospital of Jiangsu University, Department of Cardiology, Kunshan City, Jiangsu Province, China; 2 Nanjing Medical University, The First People's Hospital of Kunshan, Department of Cardiology, Gusu School, China; 3 Qilu Hospital of Shandong University, Department of Cardiology, Jinan City, China

**Keywords:** chronic heart failure, fibrin-3 (Fibulin-3), anti-human tumour endothelial marker 1, pituitary adenylate cyclase-activating polypeptide-38, cardiac function classification, hronična srčana insuficijencija, fibrin-3 (Fibulin-3), humani tumorski endotelni marker 1, hipofizni adenilat ciklažno aktivirajući peptid-38, klasifikacija srčane funkcije

## Abstract

**Background:**

To analyse the relationships between the levels of serum fibrinum-3 (Fibulin-3), anti-human tumour endothelial marker 1 (TEM 1), and pituitary adenylate cyclase-activating polypeptide-38 (PACAP-38) in elderly chronic heart failure (CHF) patients and the classification of cardiac function and an unfavourable prognosis.

**Methods:**

A total of 132 elderly CHF patients who were admitted to the hospital between July 2022 and July 2024 were chosen as the elderly CHF group, and 132 healthy people who were examined in the hospital during that time were selected as the control group. According to the cardiac function classification criteria of the New York Heart Association (NYHA), Grade II (42 patients), Grade III (51 patients), and Grade IV (39 patients) groups were created from the elderly CHF patients. Based on whether MACE happened after discharge, elderly CHF patients were split into two groups: those who experienced MACE and those who did not. The levels of serum Fibulin-3, TEM1 and PACAP-38 in all the subjects were detected via ELISA. Serum Fibulin-3, TEM 1, and PACAP-38 levels were correlated with cardiac function categorisation in older CHF patients using Spearman correlation analysis. Older patients with CHF had their MACEs examined using multivariate logistic regression analysis. Serum Fibulin-3, TEM 1, and PACAP-38 were evaluated for their predictive value of MACEs in older individuals with CHF.

**Results:**

While the elderly CHF group's serum levels of Fibulin-3 and PACAP-38 were lower than those of the control group, their serum levels of TEM1 were higher. P&lt;0.05 indicated that the differences were statistically significant. Serum TEM1 levels are compared across various cardiac function categories in elderly CHF patients. Serum Fibulin-3 and PACAP-38 levels in older CHF patients with varying cardiac function grades were compared, revealing the following pattern: Grade II &gt; Grade III &gt; Grade IV ! Spearman correlation analysis revealed a favourable correlation between the serum TEM1 level and the heart function classification of elderly CHF patients (rs=0.488, P&lt;0.05), as well as between Fibulin-3 and PACAP-38 (rs=-0.463, -0.432, P&lt;0.05). The MACE group had lower levels of serum Fibulin-3 and PACAP-38 compared to the non-MACE group, while the levels of serum TEM1 and the proportion of patients with cardiac function grade IV were higher than in the non-MACE group. Multivariate logistic regression analysis revealed that elevated levels of serum Fibulin-3 and PACAP-38 were protective factors against MACEs. In contrast, elevated levels of serum TEM1 and cardiac function grade IV were risk factors for MACEs. According to the ROC curve study, the areas under the curve (AUCs) of serum Fibulin-3, TEM1, and PACAP-38 alone and the combination of the three indicators for predicting MACEs in patients with CHF were 0.666, 0.636, 0.641, and 0.798, respectively. The AUC predicted by the combination of the three indicators was greater than that of serum Fibulin-3, TEM1, and PACA. The AUC predicted by P-38 alone (Z3 combined with Fibulin-3=2.448, P=0.014; Z3 combined with TEM 1=2.033, P=0.042; Z3 combined with PACAP-38=2.200, P=0.028).

**Conclusions:**

Serum Fibulin-3 and PACAP-38 levels were considerably lower in older CHF patients, whereas the level of TEM1 was significantly increased. These three factors together demonstrated a comparatively high predictive value for MACE occurrence in older CHF patients.

## Abstract (sr)

**Uvod:**

Cilj e ispita povezanost nivoa serumskogfibrulina-3 (Fibulin-3), humanog tumorskog endotelnogmarkera 1 (TEM 1) i hipofiznog adenilata ciklazno aktivirajućeg peptida-38 (PACAP-38) kod starijih pacijenata sahroničnom srčanom insuficijencijom (CHF) u vezi sa klasifikacijom srčane funkcije i rizikom od nepovoljnih kardiovaskularnih događaja (MACE).

**Metode:**

U studiju je uključeno ukupno 132 starija pacijenta sa CHF koji su hospitalizovani u periodu od jula 2022.do jula 2024. godine (grupa starijih sa CHF), kao i 132zdrave osobe pregledane u istom periodu (kontrolnagrupa). Prema klasifikaciji funkcionalne sposobnosti srcapo kriterijumima Njujorškog udruženja za srce (NYHA),pacijenti sa CHF podeljeni su u tri grupe: II stepen (42pacijenta), III stepen (51 pacijent) i IV stepen (39 pacijenata). Na osnovu pojave velikih neželjenih kardiovaskularnihdogađaja (MACE) nakon otpusta, pacijenti su dalje podeljeni u dve grupe: sa i bez MACE događaja. Nivoi serumskog Fibulina-3, TEM1 i PACAP-38 su određivani ELISAmetodom. Korelacija između nivoa ovih biomarkera i klasifikacije srčane funkcije je analizirana Spirmanovom korelacionom analizom. Za procenu povezanosti nivoa serumskihmarkera sa pojavom MACE događaja korišćena je multivarijantna logistička regresiona analiza. Prediktivna vrednost Fibulina-3, TEM1 i PACAP-38 za MACE procenjena jepomocu ROC krivih.

**Rezultati:**

Stariji pacijenti sa CHF su imali niže serumskenivoe Fibulina-3 i PACAP-38, a vise nivoe TEM1 upoređenju sa kontrolnom grupom (P&lt; 0,05). Nivoi serumskog TEM1 su značajno rasli sa pogorsanjem srčanefunkcije, dok su nivoi Fibulina-3 i PACAP-38 opadali (II &gt;III &gt; IV stepen). Spearmanova analiza pokazala je pozitivnukorelaciju između TEM1 i stepena srčane insuficijencije(rs= 0,488, P&lt; 0,05), kao i negativnu korelaciju izmeđuFibulina-3 i PACAP-38 i stepena srčane funkcije (rs=-0,463, rs=-0,432, P&lt; 0,05). U MACE grupi su zabeleženiniži nivoi Fibulina-3 i PACAP-38, dok su nivoi TEM1 i udeopacijenata sa IV stepenom srčane funkcije bili visi nego une-MACE grupi. Multivarijantna regresiona analiza jepokazala da su visi nivoi Fibulina-3 i PACAP-38 zastitni faktori, dok su poviseni nivoi TEM1 i IV stepen srčane funkcijerizični faktori za MACE događaje. ROC analiza je pokazalada su povrsine ispod krive (AUC) za pojedinačne markereFibulin-3, TEM1 i PACAP-38 iznosile 0,666, 0,636 i0,641, dok je kombinovana prediktivna vrednost tri markera bila visa (A U C = 0,798), sto ukazuje na vecu dijagnostičku preciznost kombinacije biomarkera.

**Zaključak:**

Kod starijih pacijenata sa hroničnom srčanominsuficijencijom nivoi serumskog Fibulina-3 i PACAP-38 suznačajno sniženi, dok je nivo TEM1 povisen. Kombinovanaanaliza ova tri biomarkera pokazuje visoku prediktivnu vrednost za pojavu velikih neželjenih kardiovaskularnih događaja (MACE).

## Introduction

Chronic heart failure (CHF) is a disorder of the heart's circulatory function [1]. The pumping function of the heart is permanently impaired. It is more common in elderly individuals and has a relatively high mortality rate. Patients with CHF have a poor overall prognosis, are prone to various adverse cardiac events, and have a relatively high mortality rate [2][3][4]. Therefore, identifying biomarkers related to the classification of cardiac function and the poor prognosis of elderly CHF patients is highly important for accurately assessing their cardiac function status, providing timely treatment, and improving survival rates [5]. Fibulin-3 is an extracellular matrix glycoprotein and is related to the formation of vascular endothelial networks [6][7][8]. Anti-human tumour endothelial marker 1 (TEM1) is a transmembrane glycoprotein that binds to cells and is upregulated in renal fibrosis, liver fibrosis and wound healing [9]. A multipurpose neuropeptide is pituitary adenylate cyclase-activating polypeptide-38 (PACAP-38) that can play a role in cardiac protection and can inhibit myocardial fibrosis. At present, there are relatively few relevant studies [10][11][12] on Fibulin-3, TEM1 and PACAP-38 in elderly patients with CHF.

The progressive illness known as chronic heart failure (CHF) is typified by neuroendocrine activity and myocardial remodelling. Its pathological process involves multiple mechanisms such as myocardial fibrosis, inflammatory response and vascular dysfunction. Accurately assessing cardiac function and prognosis remains a problematic point in clinical management. The tumour endothelial marker TEM1, a key mediator of inflammatory vascular remodelling, is significantly upregulated in the microenvironment of heart failure. Although the three have been confirmed to be related to cardiovascular injury, the dynamic change pattern of their serum levels in CHF, their correlation with cardiac function indicators (such as LVEF, NT-proBNP), and their synergistic predictive value for prognosis have not yet been revealed. To jointly detect the levels of serum PACAP-38, Fibulin-3 and TEM1 in patients with CHF, and analyse their multiple correlations with the severity of cardiac function, rehospitalisation rate and cardiovascular mortality. By clarifying the role of the fibrosis-inflammation-vascular injury network jointly involved by the three in the progression of CHF, it is expected to establish a new multi-dimensional biomarker combination, break through the limitations of traditional single-index prediction, dynamic monitoring of the disease process and development of targeted intervention strategies, and have essential translational significance for improving the precise management of CHF.

## Materials and methods

### Research subjects

One hundred thirty-two elderly CHF patients who were admitted to our hospital between July 2022 and July 2024 made up the old CHF group, while 132 healthy individuals who underwent physical examinations at our hospital during the same time period made up the control group. There were sixty-eight men and sixty-four women in the senior CHF group. The average age was 68.35±4.56 years, the body mass index (BMI) was 21.75±2.26 kg/m^2^, the systolic blood pressure was 122.59±9.71 mmHg, and the diastolic blood pressure was 71.26±7.85 mmHg. There were 36 cases of alcohol consumption, with a BMI of 21.53±2.05 kg/m^2^, a systolic blood pressure of 121.84±9.46 mmHg, and a diastolic blood pressure of 71.52±7.47 mmHg. 38 patients consumed alcohol; 45 patients smoked. There was no statistically significant difference in the general data, such as sex and age, between the two groups (P>0.05), making them comparable.

Inclusion criteria: (1) Compared with the relevant diagnostic criteria for CHF in the »International Guidelines for the Diagnosis and Treatment of Heart Failure 2022«; (2) Complete clinical data; (3) Aged 60 years. Exclusion criteria: (1) had haematological diseases; (2) had mental illness; (3) had cancerous tumours along with severe liver and renal impairment.

All research participants or their families provided informed consent, and the study was evaluated and approved by our hospital's Medical Ethics Committee (No. SDQLH-2022-045).

### Detection of serum Fibulin-3, TEM1 and PACAP-38 levels

Three to five millilitres of fasting peripheral venous blood were extracted from all patients and healthy individuals the morning after admission, and from those undergoing physical examinations on the day of the examination. The levels of serum Fibulin-3, TEM1 and PACAP-38 were detected via ELISA. Shanghai Biyun Tian Biotechnology Co., Ltd. supplied the TEM1 kit, while Abnova Company in the US provided the Fibulin-3 kit. From Beijing Dongge Boye Biotechnology Co., Ltd., the PACAP-38 kit was acquired. The test was conducted strictly in compliance with the kit's instructions. The absorbance was detected using an enzyme-labelled instrument (purchased from Shanghai Meigu Molecular Instrument Co., Ltd.), and the levels of serum Fibulin-3, TEM1, and PACAP-38 were calculated based on the standard regression curve.

### Laboratory biochemical testing methods

This study adopted the principle of the double antibody sandwich method of Enzyme-Linked Immunosorbent Assay (ELISA). Quantitative detection of the concentrations of pituitary adenylate cyclase-activating polypeptide-38 (PACAP-38), Fibulin-3 and tumour endothelial marker 1 (TEM1) in the serum of patients with chronic heart failure. This method is based on solid-phase carriers (such as 96-well plates) pre-coated with specific capture antibodies, which can capture the target antigens (PACAP-38, Fibulin-3 or TEM1) in the serum samples to be tested. After cleaning to remove unbound substances, a specific detection antibody (usually biotin-labelled) that binds to another epitope of the target antigen is added to form a »capture antibody-antigen-detection antibody« complex. Subsequently, horseradish peroxidase (HRP)-labelled Streptavidin-HRP is added, which binds with high affinity to the biotin-labelled detection antibody. After washing again, add the colour-developing substrate solution of HRP (such as 3,3',5,5' -tetramethylbenzidine, TMB). The optical density (OD value) of each well was measured at a specific wavelength (such as 450 nm) using a microplate reader.

The correlation coefficient (R^2^) of the standard curve must be greater than 0.99 to ensure quantitative accuracy. The final obtained concentration data of serum PACAP-38, Fibulin-3 and TEM1 will be correlated with the cardiac function indicators and prognosis information of the patients for analysis.

### Testing instruments and reagents

(1) The determination of serum PACAP-38 concentration was conducted using the Human PACAP-38 ELISA Kit (item No.: EK-060-08) produced by Phoenix Pharmaceuticals (located in Belmont, California, USA).

(2) The determination of serum Fibulin-3 concentration was carried out using the Human Fibulin-3 (FBLN3) ELISA Kit (catalogue number: SEA171Hu) produced by Wuhan Yunkelong Technology Co., LTD. (Wuhan, China).

(3) The determination of serum TEM1 (Endosialin/CD248) concentration was carried out using the Human Endosialin (TEM1) ELISA Kit (product number: CSB-EL027447HU) produced by Wuhan Huamei Bioengineering Co., LTD. (CUSABIO brand, Wuhan, China).

### Prognostic follow-up

Through telephone or outpatient follow-up, the occurrence of MACE (including rehospitalisation due to heart failure, myocardial infarction, death, etc.) three months after the patient's discharge was recorded. Depending on whether MACE happened after discharge.

### Statistical methods

SPSS 25.0 statistical software was used to examine the data. The notation x̄±s represents measurement data with a normal distribution. Comparisons between two groups were conducted using independent sample t tests; comparisons between multiple groups were conducted using oneway analysis of variance, and pairwise comparisons were performed using the SNK-q test. The factors impacting MACEs in older individuals with CHF were examined using multivariate logistic regression analysis. Using a receiver operating characteristic (ROC) curve, the predictive value of MACEs in elderly CHF patients was evaluated using serum Fibulin-3, TEM1, and PACAP-38.

## Results

### Comparison of serum Fibulin-3, TEM1 and PACAP-38 levels between the elderly CHF group and the control group

While the elderly CHF group had higher serum TEM1 levels than the control group, the latter group had lower levels of Fibulin-3 and PACAP-38 (P<0.05) (Table 1).

**Table 1:** Comparison of serum Fibulin-3, TEM1 and PACAP-38 levels between the elderly CHF group and the control group.

Group	n	Fibulin-3 (ng/mL)	TEM1 (ng/mL)	PACAP-38 (pg/mL)
Control group	132	65.38±16.64	1.27±0.23	112.35±20.12
Elderly CHF group	132	52.72±13.70	2.06±0.47	84.42±16.56
t		6.748	-17.346	12.314
P		≤0.001	<0.001	<0.001

By comparing the serum marker test results of 132 elderly patients with chronic heart failure (CHF) and 132 healthy controls, it was found that there were significant differences in the levels of Fibulin-3, TEM1 and PACAP-38 between the two groups. The concentrations of serum Fibulin-3 and PACAP-38 in the elderly CHF patient group were significantly lower than those in the healthy control group, with statistically significant differences between the groups (P<0.05). The serum TEM1 level in the elderly cHf group showed a significant upward trend compared with the healthy control group (P<0.05). Serum Fibulin-3 and PACAP-38 are expressed at low levels in elderly patients with CHF, while TEM1 is expressed at high levels. This suggests that these three biomarkers may jointly be involved in the pathophysiological process of chronic heart failure, and changes in their levels are closely related to the occurrence of the disease.

### Comparison of serum Fibulin-3, TEM1 and PACAP-38 levels in elderly CHF patients with different cardiac function grades

Comparison of serum TEM1 levels in elderly CHF patients with different cardiac function grades: Grade II group < Grade III group < Grade IV group. Moreover, comparisons between any two groups revealed statistically significant differences (P<0.05). A comparison of the serum Fibulin-3 and PACAP-38 levels in elderly CHF patients with different cardiac function grades revealed the following trend: Grade II > Grade III > Grade IV and the differences between any two groups were statistically significant (P<0.05) (Table 2).

**Table 2:** Serum Fibulin-3, TEM1, and PACAP-38 levels in elderly CHF patients with different cardiac function grades.

Group	n	Fibulin-3 (ng/mL)	TEM1 (ng/mL)	PACAP-38 (pg/mL)
Group II	42	58.38±14.64	1.82±0.42	91.63±17.26
Group	51	52.62±13.58	2.08±0.46	84.45±16.53
Group IV	39	46.77±12.83	2.28±0.53	76.63±15.86
F		7.248	9.794	8.282
P		<0.001	<0.001	<0.001

### Correlation analysis of cardiac function classification and serum Fibulin-3, TEM1, and PACAP-38 levels in elderly patients with CHF

Spearman correlation analysis revealed that the cardiac function classification of elderly patients with CHF was positively correlated with the serum TEM1 level (rs = 0.488, P<0.05) and had a negative correlation with PACAP-38 and Fibulin-3 levels (rs = -0.463, -0.432, P<0.05). The above results suggest that the progressive decrease of serum Fibulin-3 and PACAP-38 and the gradual increase of TEM1 are closely related to the degree of cardiac function impairment in elderly patients with CHF, and the three can be used as potential biological indicators for evaluating the severity of cardiac function.

### Data comparison between the MACE and non-MACE groups

The MACE group consisted of 38 patients, while the non-MACE group included 94 patients. The percentage of men, age, length of illness, heart rate, primary disease, and medication use did not change statistically significantly between the MACE and non-MACE groups (P>0.05). The serum TEM1 level and the proportion of patients with grade IV cardiac function classification were both significantly greater than those in the non-MACE group (P<0.05) (Table 3).

**Table 3:** Data of MACE group and non-MACE group (n(%) or x̄ ± s).

Group	n	Male		Age (Years)	Course of the<br>disease (Years)	Heart rate<br>(Beats/min)
MACE Group	38	24 (63.16)		66.41±3.56	6.37±2.61	74.43±4.65
Nonmace group	94	58 (61.70)		66.53±3.67	6.93±2.27	74.22±4.46
x^2^/t		0.024		0.172	1.228	0.242
P		0.876		0.864	0.222	0.809
Group	n	Primary disease				
		Congenital heart<br>disease		Dilated<br>cardiomyopathy	Coronary heart<br>disease	Other diseases
MACE Group	38	3 (7.89)		11 (28.95)	23 (60.53)	1(2.63)
Nonmace group	94	2 (2.13)		25 (26.60)	67 (71.28)	2(2.13)
x^2^/t		2.934				
P		0.402				
Group	n	Therapeutic drugs				
		Diuretic		Beta-blocker AC	EI/ARB class drugs	Digitalis drugs
MACE Group	38	33 (86.84)		31 (81.58)	29 (76.32)	20(52.63)
Nonmace group	94	78 (82.98)		74 (78.72)	67 (71.28)	53(56.38)
x^2^/t		0.302		0.136	0.346	0.154
P		0.583		0.712	0.556	0.695
Group	n	NYHA classification		Fibulin-3	TEM1	PACAP-38
		Grade II ~ III	Grade IV	(pg/mL)	(ng/mL)	(ng/mL)
MACE Group	38	8 (21.05)	30 (78.95)	44.28±12.94	2.37±0.49	75.62±17.05
Nonmace group	94	85 (90.43)	9 (9.57)	56.13±14.01	1.94±0.46	87.54±16.36
x^2^/t		62.563		4.495	-4.772	3.745
P		≤0.001		≤0.001	<0.001	<0.001

### Analysis of multivariate logistic regression for MACEs in older CHF patients

Multivariate logistic regression analysis was conducted with MACE occurrence in elderly patients with CHF (occurrence = 1, nonoccurrence =0) as the dependent variable and serum Fibulin-3 (original value input), TEM1 (original value input), PACAP-38 (original value input), and cardiac function classification (grade IV=1, grades II-III =0) as independent variables. Elevated levels of serum Fibulin-3 and PACAP-38 were protective factors against MACEs (P<0.05), whereas in older CHF patients, increased levels of cardiac function grade IV and serum TEM1 were risk factors for MACEs (P<0.05) (Table 4).

**Table 4:** Analysis of multivariate logistic regression for MACE in elderly CHF patients.

Factor	β	SE	Waldx^2^	P	OR	OR95%CI
NYHA classification	1.046	0.389	7.224	0.007	2.846	1.327 6.098
Fibulin-3	-0.285	0.124	5.283	0.022	0.752	0.590 0.959
TEM1	0.681	0.274	6.179	0.013	1.976	1.155 3.381
PACAP-38	-0.149	0.063	5.556	0.018	0.862	0.762 0.975

### Predictive value of serum Fibulin-3, TEM1 and PACAP-38 for the occurrence of MACEs in elderly patients with CHF

**Figure 1:**
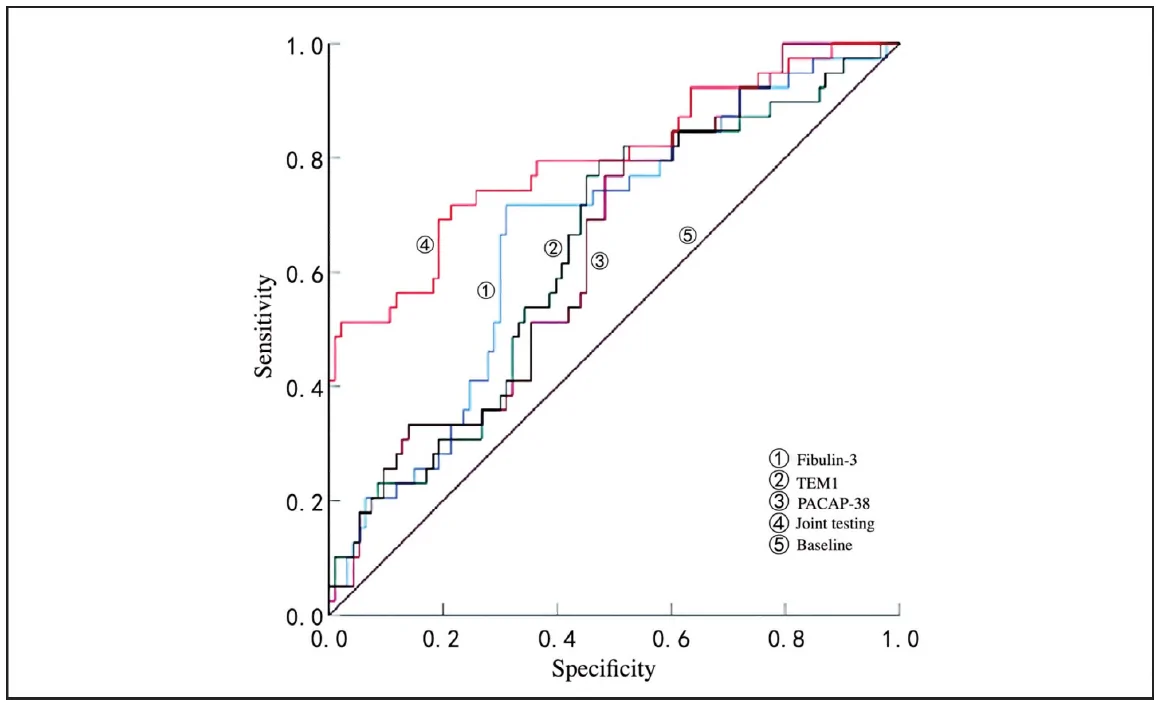
ROC curves of serum Fibulin-3, TEM1 and PACAP-38 regarding MACEs' incidence in elderly CHF patients.

ROC curve analysis was conducted with the MACE group as the positive control and the non-MACE group as the negative control. The area under the curve (AUC) values of serum Fibulin-3, TEM1, PACAP-38 alone and the combination of the three indicators for predicting MACEs in patients with CHF were 0.666, 0.636, 0.641, and 0.798, respectively. The AUC predicted by the combination of the three indicators was greater than that of serum Fibulin-3, TEM1, and PACA. The AUC predicted by P-38 alone (Z3 combined with Fibulin-3=2.448, P=0.014; Z3 combined with TEM1 =2.033, P=0.042; Z3 combined with PACAP-38=2.200, P=0.028) (Table 5 and Figure 1).

**Table 5:** The predictive value of individual and combined detection of serum Fibulin-3, TEM1, and PACAP-38 for the occurrence of MACE in elderly patients with CHF

Indicator	AUC	Optimal cutoff<br>value	AUC 95%CI	Sensitivity (%)	Specificity (%)	Youden Index	P
Fibulin-3	0.666	52.71 ng/mL	0.579 0.746	71.79	68.82	0.406	≤0.05
TEM1	0.636	2.01 ng/mL	0.548 0.718	79.49	52.69	0.322	≤0.05
PACAP-38	0.641	86.11 pg/mL	0.553 0.722	76.92	51.61	0.285	≤0.05
Three joint<br>projects	0.798	-	0.719 0.863	71.79	78.49	0.503	≤0.05

## Discussion

The heart function of elderly individuals usually decreases with age [13]. An ageing heart is more susceptible to other diseases, which in turn leads to the occurrence of CHF [14]. Current treatment methods for elderly patients with CHF include limiting sodium intake and increasing physical activity. However, if the condition is severe, cardiac surgery may be needed to improve cardiac function, which increases the incidence of poor prognosis in patients [15]. Therefore, identifying biomarkers related to the cardiac function classification and prognosis of elderly CHF patients is crucial for accurately predicting their cardiac function status, providing effective treatments, and improving their survival rate [16][17][18].

Fibulin-3 is present in various organs of the human body and is produced mainly by endothelial cells and fibroblasts in the heart. Fibulin-3 is related to diastolic blood pressure and pulse pressure, and can inhibit the expression of inflammatory factors [19]. One study [20] reported that in older hypertensive patients, the serum Fibulin-3 level was lower than in the control group. Changes in Fibulin-3 levels may lead to alterations in the vascular structure of the aortic wall. Another study revealed that the level of serum Fibulin-3 is related to the severity of CHD. The decreased level of Fibulin-3 may promote the inflammatory response and aggravate the damage to myocardial cells. Comparison of serum Fibulin-3 levels in elderly CHF patients with different cardiac function grades revealed the following trend: Grade II > Grade III > Grade IV [21]. Fibulin-3 is related to the cardiac function grade of elderly CHF patients. Silencing Fibulin-3 expression can promote the development of CHF.

TEM1 was first discovered on the surface of cancer stromal cells [22]. The expression of TEM1 increases in cardiomyocytes cultured in heart failure and hypoxia-induced conditions [23]. Comparisons of serum TEM1 levels in elderly CHF patients with different cardiac function grades show that these levels are related to the patients' cardiac function grades. That upregulation of TEM1 expression can promote the development of CHF [24][25][26]. One possible reason is that TEM1 promotes myocardial fibrosis, inhibits the regeneration and repair of myocardial cells, and increases myocardial damage [27].

PACAP-38 is a protective factor that initiates antioxidant, antiapoptotic and anti-inflammatory effects during acute cell injury [28]. One study [29] revealed that, compared to healthy controls, the plasma PACAP-38 level in CHF patients is decreased, weakening the cardioprotective mechanism. PACAP-38 treatment can significantly alleviate left ventricular systolic function impairment and effectively promote the ability of myocardial cells to counteract oxidative stress-induced apoptosis [30]. The comparison of serum PACAP-38 levels in elderly CHF patients revealed that PACAP-38 is related to the cardiac func-tion grade of elderly CHF patients and that the low expression of PACAP-38 promotes the development of CHF [31][32][33]. One possible reason is that PACAP-38 can inhibit inflammation and oxidative stress responses and promote the proliferation and repair of myocardial cells [34][35][36].

Elevated levels of serum Fibulin-3 and PACAP-38 are protective factors against MACEs in elderly CHF patients (P<0.05). In contrast, elevated serum TEM1 levels and cardiac function grade IV are risk factors for MACEs in elderly CHF patients (P<0.05). More attention should be given to elderly CHF patients with abnormal levels of Fibulin-3, TEM1 and PACAP-38. The therapeutic effect can be improved through targeted therapy with Fibulin-3, TEM1 and PACAP-38 [37][38][39][40].

The AUCs of serum Fibulin-3, TEM1, and PACAP-38 alone and the combination of the three indicators for predicting MACEs in patients with CHF were 0.666, 0.636, 0.641, and 0.798, respectively. The AUC predicted by the combination of the three indicators was greater than that predicted by serum Fibulin-3, TEM1, and PACAP-38 alone. The measured AUC (Z3 combined with Fibulin-3=2.448, P=0.014; Z3 combined with TEM1 =2.033, P=0.042; Z3 combined with PACAP-38=2.200, P=0.028) suggested that the combination of serum Fibulin-3, TEM1, and PACAP-38 had the best predictive efficacy, preventing the occurrence of MACEs. Compared with the traditional biomarker B-type natriuretic peptide, the combined application of serum Fibulin-3, TEM1, and PACAP-38 can more comprehensively and accurately evaluate elderly CHF patients' risk of MACEs from a variety of angles, improve the accuracy of prediction, and is simple to perform with strong repeatability.

## Conclusion

Serum levels of TEM1 were noticeably increased in elderly CHF patients, whereas their levels of Fibulin-3 and PACAP-38 were much lower. The classification of heart function and patient prognosis is directly linked to these findings. In older patients with CHF, the occurrence of MACEs can be more accurately predicted when all three indications are combined.

## Dodatak

### Funding

The Construction of »Integrated Prevention and Treatment of Cardiovascular Diseases in County Areas« Based on CDSS (SKY2023028).

### Conflict of interest statement

All the authors declare that they have no conflict of interest in this work.

## References

[1] Amioka M, Sanada R, Matsumura H, Kinoshita H, Sairaku A, Morishima N, Nakano Y (2023;Jan 1). Impact of SGLT2 inhibitors on old age patients with heart failure and chronic kidney disease. Int J Cardiol.

[2] Herrmann J J, Brunner-La R H, Baltussen L E H J M, Beckers-Wesche F, Bekkers S C A M, Bellersen L, van Eck J, Hassing H, Jaarsma T, Linssen G C M, Pisters R, Sanders-van Wijk S, Verdijk M H I, Handoko M L, van der Meer P, Verbrugge F H, Januzzi J L Jr, Bayés-Genis A, Nieuwlaat R, Rodwell L, Gommans D H F, van Kimmenade R R J (2025;Jun). Liberal fluid intake versus fluid restriction in chronic heart failure: A randomized clinical trial. Nat Med.

[3] Yamamoto S, Okamura M, Akashi Y J, Tanaka S, Shimizu M, Tsuchikawa Y, Ashikaga K, Kamiya K, Kato Y, Nakayama A, Makita S, Isobe M (2024;Aug 23). Impact of Long-Term Exercise-Based Cardiac Rehabilitation in Patients With Chronic Heart Failure: A Systematic Review and Meta-Analysis. Circ J.

[4] Sachdev V, Sharma K, Keteyian S J, Alcain C F, Desvigne-Nickens P, Fleg J L, Florea V G, Franklin B A, Guglin M, Halle M, Leifer E S, Panjrath G, Tinsley E A, Wong R P, Kitzman D W, American Heart Association Heart Failure and Transplantation Committee of the Council on Clinical Cardiology, Council on Arteriosclerosis, Thrombosis and Vascular Biology, American College of Cardiology (2023;Apr 18). Supervised Exercise Training for Chronic Heart Failure With Preserved Ejection Fraction: A Scientific Statement From the American Heart Association and American College of Cardiology. Circulation.

[5] Quiroga B, Ortiz A, Núñez S, Kislikova M, González-Sanchidrián S, Broseta J J, Albines Z S, Escamilla-Cabrera B, Rivero Viera V, Rodriguez Santarelli D, Salanova-Villanueva L, Lopez-Rodriguez F, Cancho-Castellano B, Ibánez-Cerezon M, Gutierrez Rivas C P, Aresté N, Campos-Gutiérrez B, Rodenas-Gálvez A, Glucksmann-Pizá M C, Balda-Manzanos S, Soldevila A, Rodriguez Gayo L, Moral-Berrio E, Ortega-Diaz M, Beltrán-Catalán S, Puente-Garcia A, Angel-Rojas M, Sosa-Barrios R H, Santana-Zapatero H, Rangel-Hidalgo G, Martinez-Canet A M, Diez J, Cardiorenal Medicine Group (CaReSEN) of the Spanish Society of Nephrology (2024). Treatment of chronic heart failure in advanced chronic kidney disease: The HAKA multicenter retrospective real-world study. Cardiorenal Med.

[6] Li Y, Xu C, Qin Z, Ge L (2024;Dec 10). Relationship Between the Hemoglobin-to-Red Cell Distribution Width Ratio and in-Hospital Mortality in Patients with Chronic Heart Failure. Vasc Health Risk Manag.

[7] Lai Y, Lin C, Liu X, Liu Y, Cai H, Zhao N, Gao Y, Yi Z, Huang J, Li M, Xu L (2025;Mar 21). Association of triglyceride-glucose index trajectories with the risk of worsening heart failure in elderly patients with chronic heart failure and type 2 diabetes: A competing risk analysis. Cardiovasc Diabetol.

[8] Rao V N, Hellkamp A S, Thomas L E, Fonarow G C, Fiuzat M, O.Connor C M, Spertus J A, Desai A S, Albert N M, Butler J, Hernandez A F, Devore A D (2024;Nov). Optimal Medical Therapy and Outcomes Among Patients With Chronic Heart Failure With Reduced Ejection Fraction. JACC Heart Fail.

[9] Kirkopoulos A, M'pembele R, Roth S, Stroda A, Larmann J, Gillmann H J, Kotfis K, Ganter M T, Bolliger D, Filipović M, Guzzetti L, Mauermann E, Ionescu D, Spadaro S, Szczeklik W, de Hert S, Beck-Schimmer B, Howell S J, Lurati-Buse G A, METREPAIR Investigators (2025;Aug). Outcomes in patients with chronic heart failure undergoing noncardiac surgery: A secondary analysis of the METREPAIR international cohort study. Anaesthesia.

[10] Cheng Y, Peng Q, Ding H, Hu M, Li C (2024чOct 25). Pathway analysis of the impact of health literacy, social support, and self-management on frailty in patients with chronic heart failure: A cross-sectional study. Medicine (Baltimore).

[11] Gayán-Ordás J, Nuñez J, Bascompte Claret R, Llacer P, Zegri-Reiriz I, de la Espriella R, Fort A, Rubio-Gracia J, Blazquez-Bermejo Z, Mendez A, Ponz I, Rodriguez-Chaverri A, Caravaca-Pérez P, Recio-Mayoral А, Jiménez-Rubio C, Pomares A, José-Soler M, Fluvia P, Garcia-Magallon B, Luis-Gorriz J, Manzano L, Husain-Syed F, Cobo-Marcos M (2024). Usefulness of Antigen Carbohydrate 125 and N-Terminal Pro-B-Type Natriuretic Peptide for Assessing Congestion in Chronic Heart Failure: Insights from the CARDIOREN Registry. Cardiorenal Med.

[12] Yan W, Tang H, Yang Y, He K (2023;Nov 30). Serum uric acid and outcome in hospitalized elderly patients with chronic heart failure through the whole spectrum of ejection fraction phenotypes. BMC Cardiovasc Disord.

[13] Wu L, Zheng Y, Liu J, Luo R, Wu D, Xu P, Wu D, Li X (2021;Apr 20). Comprehensive evaluation of the efficacy and safety of LPV/r drugs in the treatment of SARS and MERS to provide potential treatment options for COVID-19. Aging (Albany NY).

[14] Zhou C, Wang S, Sun X, Han Y, Zhang L, Liu M (2023;Feb 10). Application of food exchange portion method in home-based nutritional intervention for elderly patients with chronic heart failure. BMC Cardiovasc Disord.

[15] Militaru M, Lighezan D F, Tudoran C, Tudoran M, Militaru A G (2024;Nov 13). Factors Influencing the Development and Severity of Cognitive Decline in Patients with Chronic Heart Failure. Medicina (Kaunas).

[16] Campbell-Quintero S, Echeverría L E, Gómez-Mesa J E, Rivera-Toquica A, Rentería-Asprilla C A, López-Garzón N A, Alcalá-Hernández A E, Accini-Mendoza J L, Baquero-Lozano G A, Martfnez-Carvajal A R, Cadena A, Zarama-Márquez M H, Ramírez-Puentes E G, Bustamante R I, Saldarriaga C (2023;May 1). Comorbidity profile and outcomes in patients with chronic heart failure in a Latin American country: Insights from the Colombian heart failure registry (RECOLFACA). Int J Cardiol.

[17] Wu L, Zhong Y, Wu D, Xu P, Ruan X, Yan J, Liu J, Li X (2022;Sep 10). Immunomodulatory Factor TIM3 of Cytolytic Active Genes Affected the Survival and Prognosis of Lung Adenocarcinoma Patients by Multi-Omics Analysis. Biomedicines.

[18] Yang X, Ren S, Liu Y, Wu X, Hao X, Bai X, Li R (2023;Dec 28). Exploration on the Value of Circulation Quality Control Intervention Mode in Percutaneous Coronary Intervention in Patients with Coronary Heart Disease and Chronic Heart Failure. Heart Surg Forum.

[19] Misumi K, Matsue Y, Nogi K, Fujimoto Y, Kagiyama N, Kasai T, Kitai T, Oishi S, Akiyama E, Suzuki S, Yamamoto M, Kida K, Okumura T, Nogi M, Ishihara S, Ueda T, Kawakami R, Saito Y, Minamino T (2025). Usefulness of hypochloremia at the time of discharge to predict prognosis in patients with chronic heart failure after hospitalization. J Cardiol.

[20] Crisafulli E, Sartori G, Vianello A, Busti F, Nobili A, Mannucci P M, Girelli D, Reposi I (2023;Mar). Clinical features and outcomes of elderly hospitalised patients with chronic obstructive pulmonary disease, heart failure or both. Intern Emerg Med.

[21] Wu L, Liu Q, Ruan X, Luan X, Zhong Y, Liu J, Yan J, Li X (2023;Jun 29). Multiple Omics Analysis of the Role of RBM10 Gene Instability in Immune Regulation and Drug Sensitivity in Patients with Lung Adenocarcinoma (LUAD). Biomedicines.

[22] Shang G, Gao Y, Liu K, Wang X (2023;Apr). Serum potassium in elderly heart failure patients as a predictor of readmission within 1 year. Heart Vessels.

[23] Duca Ștefania-Teodora, Tudorancea I, Haba M Ștefan C, Costache A D, Șerban Ionela-Lăcrămioara, Pavăl D, Loghin C, Costache-Enache I (2024;Aug). Enhancing Comprehensive Assessments in Chronic Heart Failure Caused by Ischemic Heart Disease: The Diagnostic Utility of Holter ECG Parameters. Medicina (Kaunas).

[24] Zhao Y, Pang M, Xu Y (2024;Sep 13). CICARE communication model and hierarchical responsibility nursing coordination in the application research of elderly patients with chronic heart failure. Medicine (Baltimore).

[25] Wu L, Zheng Y, Ruan X, Wu D, Xu P, Liu J, Wu D, Li X (2022;Jan 1). Long-chain noncoding ribonucleic acids affect the survival and prognosis of patients with esophageal adenocarcinoma through the autophagy pathway: Construction of a prognostic model. Anticancer Drugs.

[26] Yuecel G, Gaasch L, Kodeih A, Hetjens S, Yazdani B, Pfleger S, Duerschmied D, Abraham W T, Akin I, Kuschyk J (2025;Feb). Device-Therapy in Chronic Heart Failure: Cardiac Contractility Modulation Versus Cardiac Resynchronization Therapy. ESC Heart Fail.

[27] Wu L, Zhong Y, Yu X, Wu D, Xu P, Lv L, Ruan X, Liu Q, Feng Y, Liu J, Li X (2022;Oct 1). Selective poly adenylation predicts the efficacy of immunotherapy in patients with lung adenocarcinoma by multiple omics research. Anticancer Drugs.

[28] Ališauskas A, Dzikevičiūtė K, Rimšaitė U, Naudžiūnas A, Razvadauskas H, Zinkienė D, Repečka T, Jucevičius J, Sadauskas S (2024;Sep 27). Prognostic Value of Transthoracic Impedance Cardiography, Amino-Terminal Pro-B-Type Natriuretic Peptide Levels, The Six-Minute Walk Test, and Chest X-Ray in Elderly Patients with Chronic Heart Failure: A Comparative Study in Lithuania. Med Sci Monit.

[29] Meng Y, Zhang T, Ge X, Zheng Q, Feng T (2024;Apr 30). Physical activity changes and related factors in chronic heart failure patients during the postdischarge transition period: A longitudinal study. BMC Cardiovasc Disord.

[30] Szymczak A, Skwarek-Dziekanowska A, Sobieszek G, Małecka-Massalska T, Powrózek T (2025;Jun 15). Comparison of the clinical value of inflammatory blood biomarkers in relation to disease severity and survival in chronic heart failure. Int J Cardiol.

[31] Armentaro G, Cassano V, Magurno M, Pastura C A, Divino M, Severini G, Martire D, Miceli S, Maio R, Mazza E, Montalcini T, Pujia A, Sciacqua A (2025;May 19). Gliflozines as add-on to Arni in echocardiographic, sarcopenic and oxidative stress parameters in elderly patients with chronic heart failure. Aging Clin Exp Res.

[32] Wu L, Li H, Liu Y, Fan Z, Xu J, Li N, Qian X, Lin Z, Li X, Yan J (2024). Research progress of 3D-bioprinted functional pancreas and in vitro tumor models. International Journal of Bioprinting.

[33] Estler B, Fröhlich H, Täger T, Hund H, Frey N, Luft T, Frankenstein L (2025;Nov 1). Endothelial dysfunction assessed by the endothelial activation and stress index (EASIX) predicts risk of mortality in chronic heart failure patients. Int J Cardiol.

[34] Ujiro S, Fujimoto W, Takemoto M, Kuroda K, Yamashita S, Imanishi J, Iwasaki M, Todoroki T, Nagao M, Konishi A, Shinohara M, Toh R, Nishimura K, Okuda M, Otake H (2025;Mar 25). Impact of Cardiorenal Anemia Syndrome on the Prognosis of Patients With Chronic Heart Failure in Japan: Insights From the KUNIUMI Registry Chronic Cohort. Circ J.

[35] Chen G, Zhang Y, Wu P (2024;Sep). Study on the application effect of nursing intervention based on the transtheoretical model in the rehabilitation treatment of patients with chronic heart failure. Altern Ther Health Med.

[36] Wu L, Chen X, Zeng Q, Lai Z, Fan Z, Ruan X, Li X, Yan J (2024;Mar 28). RETRACTED: NR5A2 gene affects the overall survival of LUAD patients by regulating the activity of CSCs through SNP pathway by OCLR algorithm and immune score. Heliyon.

[37] Chen L, Xu F, Tong Q, Wang G (2024;Dec 13). Blood routine test-based biomarkers is related to bone mineral density in elderly patients with chronic heart failure: A retrospective study. Medicine (Baltimore).

[38] Wu L, Li X, Qian X, Wang S, Liu J, Yan J (2024;Feb 12). Lipid Nanoparticle (LNP) Delivery Carrier-Assisted Targeted Controlled Release mRNA Vaccines in Tumor Immunity. Vaccines (Basel).

[39] Koontalay A, Botti M, Hutchinson A (2024;Oct). Illness perceptions of people living with chronic heart failure and limited community disease management. J Clin Nurs.

[40] Izraiq M, Alawaisheh R, Ibdah R, Dabbas A, Ahmed Y B, Mughrabi S A, Zuraik A, Ababneh M, Toubasi A A, Al-Bkoor B, Abu-Hantash H (2024;May 19). Machine Learning-Based Mortality Prediction in Chronic Kidney Disease among Heart Failure Patients: Insights and Outcomes from the Jordanian Heart Failure Registry. Medicina (Kaunas).

